# Comparative clinical outcomes between direct oral anticoagulants and warfarin among elderly patients with non-valvular atrial fibrillation in the CMS medicare population

**DOI:** 10.1007/s11239-019-01838-5

**Published:** 2019-03-28

**Authors:** Alpesh Amin, Allison Keshishian, Oluwaseyi Dina, Amol Dhamane, Anagha Nadkarni, Eric Carda, Cristina Russ, Lisa Rosenblatt, Jack Mardekian, Huseyin Yuce, Christine L. Baker

**Affiliations:** 1 Department of Medicine, University of California, 101 The City Drive South, Building 26, Room 1000, ZC-4076H, Orange, CA 92868 USA; 2 STATinMED, Ann Arbor, MI USA; 3 Pfizer Inc., New York, NY USA; 4 Bristol-Myers Squibb Company, Lawrenceville, NJ USA; 5 New York City College of Technology, City University of New York, New York, NY USA

**Keywords:** Apixaban, Dabigatran, Rivaroxaban, Warfarin, Non-valvular atrial fibrillation, Medicare

## Abstract

Atrial fibrillation (AF) prevalence increases with age; > 80% of US adults with AF are aged ≥ 65 years. Compare the risk of stroke/systemic embolism (SE), major bleeding (MB), net clinical outcome (NCO), and major adverse cardiac events (MACE) among elderly non-valvular AF (NVAF) Medicare patients prescribed direct oral anticoagulants (DOACs) vs warfarin. NVAF patients aged ≥ 65 years who initiated DOACs (apixaban, dabigatran, and rivaroxaban) or warfarin were selected from 01JAN2013-31DEC2015 in CMS Medicare data. Propensity score matching was used to balance DOAC and warfarin cohorts. Cox proportional hazards models estimated the risk of stroke/SE, MB, NCO, and MACE. 37,525 apixaban–warfarin, 18,131 dabigatran–warfarin, and 55,359 rivaroxaban–warfarin pairs were included. Compared to warfarin, apixaban (HR: 0.69; 95% CI 0.59–0.81) and rivaroxaban (HR: 0.82; 95% CI 0.73–0.91) had lower risk of stroke/SE, and dabigatran (HR: 0.88; 95% CI 0.72–1.07) had similar risk of stroke/SE. Apixaban (MB: HR: 0.61; 95% CI 0.57–0.67; NCO: HR: 0.64; 95% CI 0.60–0.69) and dabigatran (MB: HR: 0.79; 95% CI 0.71–0.89; NCO: HR: 0.84; 95% CI 0.76–0.93) had lower risk of MB and NCO, and rivaroxaban had higher risk of MB (HR: 1.08; 95% CI 1.02–1.14) and similar risk of NCO (HR: 1.04; 95% CI 0.99–1.09). Compared to warfarin, apixaban had a lower risk for stroke/SE, MB, and NCO; dabigatran had a lower risk of MB and NCO; and rivaroxaban had a lower risk of stroke/SE but higher risk of MB. All DOACs had lower risk of MACE compared to warfarin.

**Electronic supplementary material:**

The online version of this article (10.1007/s11239-019-01838-5) contains supplementary material, which is available to authorized users.

## Highlights

- The prevalence of NVAF and risk of stroke increase with age.
- Few studies have compared DOACs to warfarin among elderly NVAF patients regarding such outcomes.
- This study showed that compared to warfarin, all DOACs were associated with lower risk of MACE, and there were varying rates of stroke/SE, MB, and NCO between the individual DOACs and warfarin.
- The findings warrant more studies to better understand effectiveness and safety profiles in the elderly NVAF population.

## Introduction

The 2010 Global Burden of Disease Study estimated the worldwide age-adjusted prevalence of atrial fibrillation (AF) at 596 per 100,000 men and 373 per 100,000 women, equating to 33.5 million individuals (20.9 and 12.6 million men and women, respectively) [1]. In the United States, the estimated prevalence of AF is 3–5 million [2, 3]. The proportion of AF patients was found to increase sharply with age, especially in people aged ≥ 65 years, who account for three-quarters of the AF population [3].

Patients with AF diagnoses are at a nearly fivefold greater risk of stroke [4]. Moreover, the AF-attributable risk for ischemic stroke is age-dependent and increases from 4.6 to 7.9% to > 10% among patients aged 50–59, 60–69, and ≥ 70 years, respectively [4]. Hence, the stroke risk stratification schema CHA_2_DS_2_-VASc score considers older age (65–74 and ≥ 75 years) as a risk factor for stroke and thromboembolism in AF patients [5].

Oral anticoagulants (OACs) prevent stroke and systemic embolism (SE) among AF patients; they are recommended by the American College of Cardiology (ACC) and the American Heart Association (AHA) guidelines for patients with non-valvular AF (NVAF) and prior stroke, transient ischemic attack (TIA), or a CHA_2_DS_2_-VASc score ≥ 2 [6]. Warfarin, a vitamin K antagonist (VKA), has been used for stroke prevention among AF patients for decades. However, the narrow therapeutic window and increased risk of bleeding have hindered use, especially among the elderly [6].

In recent years, randomized clinical trials have demonstrated that compared to warfarin, direct OACs (DOACs)—including apixaban, dabigatran, edoxaban, and rivaroxaban—were all associated with similar to lower risk of stroke/SE and major bleeding (MB) among elderly patients [7–9]. Introduced in 2008, the Fit-fOR-The-Aged (FORTA) classification is the first system with both negative (harmful or critical drugs: D and C labels) and positive (beneficial drugs: A and B labels) labelling at the individual drug and drug group levels. Based on FORTA and the Delphi process, warfarin, dabigatran, edoxaban, and rivaroxaban were labelled B (beneficial; safely and effectively treat AF), and apixaban was labeled A (absolutely; most beneficial risk–benefit ratio) for the treatment of AF patients aged > 65 years [10].

Using the largest US claims database of elderly patients, we evaluated real-world comparative risks of stroke/SE, MB, net clinical outcomes (stroke/SE or MB [NCO]), and major adverse cardiac events (MACE) among NVAF patients who initiated either DOACs (apixaban, dabigatran, and rivaroxaban) or warfarin. This study added more recent data and additional outcome measures to our previous study, which provides comprehensive and current evidence to help prevent stroke among the elderly NVAF population [11]. The results also supplement clinical trials and add key information to real-world literature.

## Methods

### Data source

This retrospective observational study used the fee-for-service (FFS) US Centers for Medicare & Medicaid Services (CMS) data from 01JAN2012-31DEC2015. This dataset is composed of adults aged ≥ 65 years, certain young people with disabilities, and people with end-stage renal disease. As of 2015, > 38 million beneficiaries were enrolled in this insurance [12]. The data include institutional (inpatient, skilled nursing facility, home health, hospice, and hospital outpatient) and non-institutional (physician/supplier–carrier and durable medical equipment) claims and Part D prescription claims, coded using International Classification of Diseases, Ninth/Tenth Revision, Clinical Modification (ICD-9/10-CM) diagnosis and procedure codes, the Health Care Common Procedure Coding System, Current Procedural Terminology codes, and National Drug Codes [13].

### Patient selection

AF (ICD-9-CM: 427.31 or ICD-10-CM: I48.0-I48.2, I48.91) patients aged ≥ 65 years with ≥ 1 pharmacy claim for apixaban, dabigatran, edoxaban, rivaroxaban, or warfarin between 01JAN2013-31DEC2015 (identification period) were selected. The first DOAC claim date during the identification period was designated as the index date for patients with any DOAC claim; the first warfarin prescription date was designated as the index date for those without a DOAC claim [14]. Patients were also required to have continuous health plan enrollment with both medical and pharmacy benefits for the 12-month pre-index (baseline) period.

**Fig. 1:**
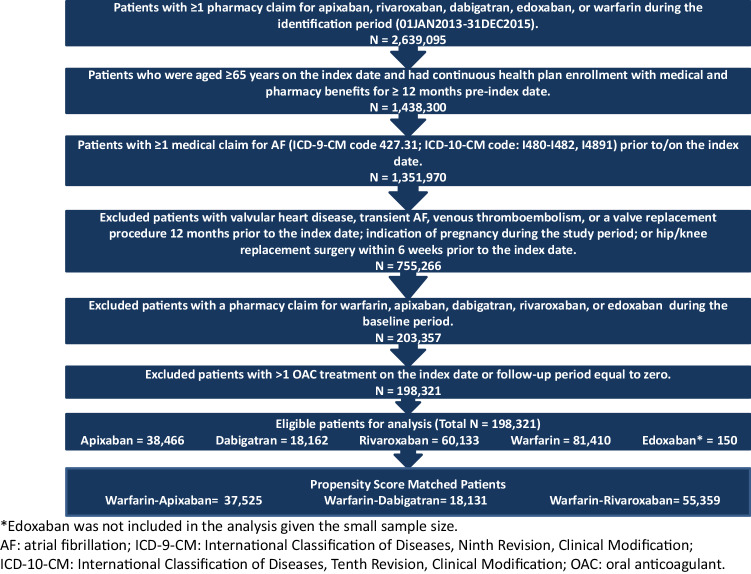
Patient selection criteria

To select OAC treatment-naïve patients, those with any OAC claim during the baseline period were excluded. Patients with evidence of valvular heart disease or transient AF during the baseline period were also excluded. To omit OAC use for the treatment or prophylaxis of venous thromboembolism (VTE), patients with VTE in the baseline period or who had hip or knee replacement surgery within 6 weeks prior to the index date were excluded. Detailed selection criteria appear in Fig. 1.

### Outcome measures

The primary outcomes were the occurrence of stroke/SE and MB, identified by hospitalizations with stroke/SE or MB as the principal diagnosis. Stroke/SE was further categorized by ischemic stroke, hemorrhagic stroke, and SE; MB was categorized by gastrointestinal (GI) bleeding, intracranial hemorrhage (ICH), and MB at other key sites [15, 16].

The secondary outcomes were NCO (a composite of stroke/SE and MB) and MACE, comprised of stroke (hemorrhagic and ischemic stroke), myocardial infarction (MI), and all-cause death. Claims databases cannot evaluate cardiovascular-related death, so the MACE definition included all-cause death.

Patients were censored at the earliest of the discontinuation date of the index treatment (no evidence of a prescription for 30 days from the last day of the index medication days’ of supply), date of switch from the index drug to another OAC (a prescription for an OAC other than the index drug within 30 days before or after the discontinuation date), date of death, end of continuous enrollment, or end of study.

### Statistical methods

One-to-one propensity score matching (PSM) was conducted between DOACs and warfarin (apixaban versus warfarin, dabigatran versus warfarin, and rivaroxaban versus warfarin) to control for potential confounders such as baseline demographics and clinical characteristics.

Using established methodology, propensity scores were generated by logistic regression. Age, sex, US geographic region, Charlson comorbidity index (CCI) [17], CHA_2_DS_2_-VASc, and HAS-BLED scores, prior bleeding and stroke, comorbidities, baseline co-medications, and baseline inpatient visits were included in the models as covariates. The nearest neighbor without replacement method and a caliper of 0.01 were implemented in the PSM [18]. After PSM, the balance of covariates was checked based on standardized differences, with a threshold of 10% [19].

For post-PSM cohorts, the incidence of primary and secondary outcomes was calculated as the number of events per 100 person-years.

Cox proportional hazards models with robust sandwich estimates were used to evaluate the hazard ratios (HRs) of stroke/SE, MB, NCO, and MACE in each matched cohort [18]. After ensuring all the matched baseline covariates were balanced post-PSM, OAC treatment was included in the Cox models as the only independent variable.

Sensitivity analysis was conducted wherein patients were censored at 6 months of follow-up, creating more balance between cohorts.

Statistical analyses were performed using the Statistical Analysis System (SAS) Version 9.3 (Cary, NC).

## Results

The study included eligible 198,171 patients; 81,410 (41.1%) were prescribed warfarin, 38,466 (19.4%) apixaban, 18,162 (9.2%) dabigatran, and 60,133 (30.3%) rivaroxaban (Fig. 1). Edoxaban was excluded due to small sample size (N = 150). Before PSM, patients who initiated warfarin were older with a mean age of 79 years, followed by those who initiated apixaban (78 years), rivaroxaban (78 years), and dabigatran (77 years). In addition, warfarin patients also had higher CCI and CHA_2_DS_2_-VASc scores than DOAC patients (Table 1).

**Table 1:** Baseline descriptive table before PSM

	Warfarin (N = 81,410)		Apixaban (N = 38,466)			Dabigatran (N = 18,162)			Rivaroxaban (N = 60,133)		
	N/mean	%/SD	N/mean	%/SD	STD^a^	N/mean	%/SD	STD^a^	N/mean	%/SD	STD^a^
*Age*	78.9	7.5	78.3	7.5	6.9	77.0	7.0	***25.0***	77.6	7.3	***16.8***
65–74	26,091	32.0%	13,627	35.4%	7.1	7479	41.2%	***19.0***	23,255	38.7%	***13.9***
75–84	35,012	43.0%	15,916	41.4%	3.3	7607	41.9%	2.3	25,119	41.8%	2.5
≥ 85	20,307	24.9%	8923	23.2%	4.1	3076	16.9%	***19.8***	11,759	19.6%	***13.0***
*Sex*											
Male	41,002	50.4%	18,581	48.3%	4.1	9338	51.4%	2.1	29,894	49.7%	1.3
Female	40,408	49.6%	19,885	51.7%	4.1	8824	48.6%	2.1	30,239	50.3%	1.3
*Race*											
White	73,714	90.5%	35,311	91.8%	4.4	16,309	89.8%	2.5	54,642	90.9%	1.1
Black	4246	5.2%	1432	3.7%	7.2	785	4.3%	4.2	2336	3.9%	6.4
Hispanic	1037	1.3%	417	1.1%	1.8	290	1.6%	2.7	931	1.5%	2.3
Other	2413	3.0%	1306	3.4%	2.5	778	4.3%	7.1	2224	3.7%	4.1
*Geographic region*											
Northeast	16,018	19.7%	6514	16.9%	7.1	3606	19.9%	0.4	10,596	17.6%	5.3
North Central	25,076	30.8%	7911	20.6%	***23.6***	4184	23.0%	***17.6***	13,341	22.2%	***19.6***
South	26,486	32.5%	17,229	44.8%	***25.4***	6953	38.3%	***12.0***	25,007	41.6%	***18.8***
West	13,745	16.9%	6791	17.7%	2.0	3387	18.6%	4.6	11,080	18.4%	4.0
Other	85	0.1%	21	0.1%	1.8	32	0.2%	1.9	109	0.2%	2.0
*Medicaid dual-eligibility*	18,908	23.2%	7488	19.5%	9.2	4268	23.5%	0.6	13,100	21.8%	3.5
*Part D low-income subsidy*	21,374	26.3%	8560	22.3%	9.3	4814	26.51%	0.6	14,734	24.5%	4.0
*Baseline comorbidity*											
Deyo-Charlson comorbidity index	3.1	2.8	2.9	2.6	9.4	2.5	2.4	***24.7***	2.7	2.5	***17.9***
CHADS2 score	2.9	1.4	2.8	1.5	6.9	2.6	1.4	***19.1***	2.7	1.4	***15.3***
CHA_2_DS_2_-VASc score	4.7	1.7	4.6	1.8	5.5	4.4	1.7	***20.2***	4.5	1.7	***14.3***
HAS-BLED score^b^	3.3	1.3	3.4	1.3	4.3	3.2	1.2	***14.1***	3.3	1.2	5.2
Baseline prior bleed	24,780	30.4%	11,807	30.7%	0.6	4731	26.0%	9.8	17,374	28.9%	3.4
Baseline prior stroke	12,496	15.3%	5280	13.7%	4.6	2159	11.9%	***10.1***	7385	12.3%	8.9
Congestive heart failure	29,326	36.0%	12,064	31.4%	9.9	5118	28.2%	***16.9***	17,287	28.7%	***15.6***
Diabetes	32,705	40.2%	13,602	35.4%	9.9	6737	37.1%	6.3	21,456	35.7%	9.3
Hypertension	71,416	87.7%	34,649	90.1%	7.5	15,964	87.9%	0.5	53,191	88.5%	2.3
Renal disease	21,021	25.8%	8599	22.4%	8.1	2892	15.9%	***24.5***	10,465	17.4%	***20.6***
Myocardial infarction	12,024	14.8%	5040	13.1%	4.8	1940	10.7%	***12.3***	7224	12.0%	8.1
Dyspepsia or stomach discomfort	17,317	21.3%	8699	22.6%	3.2	3607	19.9%	3.5	13,060	21.7%	1.1
Peripheral vascular disease	46,697	57.4%	22,742	59.1%	3.6	9689	53.3%	8.1	33,670	56.0%	2.8
Peripheral artery disease	20,131	24.7%	8932	23.2%	3.5	3635	20.0%	***11.3***	13,237	22.0%	6.4
Transient ischemic attack	6411	7.9%	3528	9.2%	4.6	1342	7.4%	1.8	4751	7.9%	0.1
Coronary artery disease	40,079	49.2%	19,962	51.9%	5.3	8367	46.1%	6.3	29,066	48.3%	1.8
*Baseline medication use*											
Angiotensin converting enzyme inhibitor	30,102	37.0%	13,194	34.3%	5.6	6875	37.9%	1.8	21,463	35.7%	2.7
Amiodarone	5612	6.9%	4300	11.2%	***15.0***	1636	9.0%	7.8	5308	8.8%	7.2
Angiotensin receptor blocker	17,030	20.9%	10,056	26.1%	***12.3***	4498	24.8%	9.2	15,149	25.2%	***10.2***
Beta blockers	42,053	51.7%	22,070	57.4%	***11.5***	9756	53.7%	4.1	32,812	54.6%	5.8
H2-receptor antagonist	5699	7.0%	2828	7.4%	1.4	1214	6.7%	1.3	4181	7.0%	0.2
Proton pump inhibitor	24,020	29.5%	13,008	33.8%	9.3	5358	29.5%	0.0	19,152	31.8%	5.1
Anti-platelets	15,589	19.1%	9235	24.0%	***11.8***	3450	19.0%	0.4	13,101	21.8%	6.5
Statins	45,149	55.5%	23,492	61.1%	***11.4***	10,476	57.7%	4.5	34,956	58.1%	5.4
*Inpatient admission*	36,572	44.9%	15,168	39.4%	***11.1***	6830	37.6%	***14.9***	24,807	41.3%	7.4

Std Difference greater than 10 is considered significant is given in bolditalic

CHA_2_DS_2_-VASc: congestive heart failure, hypertension, age ≥ 75 years, diabetes mellitus, prior stroke or transient ischemic attack or thromboembolism, vascular disease, age 65–74 years, sex category; HAS-BLED: hypertension, abnormal renal and liver function, stroke, bleeding, labile INRs (international normalized ratio), elderly, drugs, and alcohol; PSM: propensity score matching; SD: standard deviation

^a^Std Difference = 100*|actual std diff|

^b^As the INR value was not available in the data, a modified HAS-BLED score was calculated using a range of 0 to 8

Through PSM, 37,525 apixaban, 18,131 dabigatran, and 55,359 rivaroxaban patients were separately matched to warfarin patients. Baseline characteristics were balanced after matching with mean standardized differences < 10%. For the matched cohorts, the means were: age: 77–78 years, CHA_2_DS_2_-VASc scores: 4.4–4.6, and HAS-BLED scores: 3.2–3.4 (Table 2). Patient data were assessed for a mean duration of 8–10 months. 71%, 80%, and 66% of patients were prescribed the standard dose of DOAC (apixaban 5 mg, dabigatran 150 mg, and rivaroxaban 20 mg), respectively.

**Table 2:** Baseline descriptive and mean follow-up time table after PSM between warfarin and DOACs

	Apixaban–warfarin cohort				Dabigatran–warfarin cohort				Rivaroxaban–warfarin cohort			
	Apixaban		Warfarin		Dabigatran		Warfarin		Rivaroxaban		Warfarin	
	(N = 37,525)		(N = 37,525)		(N = 18,131)		(N = 18,131)		(N = 55,359)		(N = 55,359)	
	N/mean	%/SD	N/mean	%/SD	N/mean	%/SD	N/mean	%/SD	N/mean	%/SD	N/mean	%/SD
*Age*	78.4	7.5	78.4	7.4	77.1	7.0	77.3	7.1	77.9	7.3	78.0	7.3
65–74	13,136	35.0%	13,204	35.2%	7449	41.1%	7472	41.2%	20,220	36.5%	20,202	36.5%
75–84	15,614	41.6%	15,698	41.8%	7606	42.0%	7602	41.9%	23,651	42.7%	23,685	42.8%
≥ 85	8775	23.4%	8623	23.0%	3076	17.0%	3057	16.9%	11,488	20.8%	11,472	20.7%
*Sex*												
Male	18,176	48.4%	18,112	48.3%	9313	51.4%	9268	51.1%	27,463	49.6%	27,494	49.7%
Female	19,349	51.6%	19,413	51.7%	8818	48.6%	8863	48.9%	27,896	50.4%	27,865	50.3%
*Race*												
White	34,436	91.8%	34,369	91.6%	16,288	89.8%	16,308	89.9%	50,418	91.1%	50,373	91.0%
Black	1424	3.8%	1451	3.9%	785	4.3%	816	4.5%	2282	4.1%	2309	4.2%
Hispanic	412	1.1%	427	1.1%	288	1.6%	269	1.5%	788	1.4%	797	1.4%
Other	1253	3.3%	1278	3.4%	770	4.2%	738	4.1%	1871	3.4%	1880	3.4%
*Geographic region*												
Northeast	6486	17.3%	6530	17.4%	3606	19.9%	3559	19.6%	10,234	18.5%	10,215	18.5%
North central	7906	21.1%	7897	21.0%	4184	23.1%	4135	22.8%	13,233	23.9%	13,260	24.0%
South	16,433	43.8%	16,467	43.9%	6932	38.2%	7161	39.5%	21,568	39.0%	21,515	38.9%
West	6679	17.8%	6615	17.6%	3379	18.6%	3245	17.9%	10,241	18.5%	10,292	18.6%
Other	21	0.1%	16	0.0%	30	0.2%	31	0.2%	83	0.1%	77	0.1%
*Medicaid dual-eligibility*	7399	19.7%	7509	20.0%	4257	23.5%	4230	23.3%	12,157	22.0%	12,053	21.8%
*Part D low-income subsidy*	8454	22.5%	8584	22.9%	4801	26.5%	4782	26.4%	13,697	24.7%	13,620	24.6%
*Baseline comorbidity*												
Deyo-Charlson comorbidity index	2.9	2.6	2.9	2.7	2.5	2.4	2.5	2.4	2.7	2.5	2.7	2.6
CHADS_2_ score	2.8	1.5	2.8	1.4	2.6	1.4	2.6	1.4	2.7	1.4	2.7	1.4
CHA_2_DS_2_-VASc score	4.6	1.8	4.7	1.7	4.4	1.7	4.4	1.7	4.5	1.7	4.5	1.7
HAS-BLED score^a^	3.4	1.3	3.4	1.3	3.2	1.2	3.2	1.2	3.3	1.3	3.3	1.3
Baseline prior bleed	11,495	30.6%	11,455	30.5%	4726	26.1%	4748	26.2%	16,013	28.9%	16,128	29.1%
Baseline prior stroke	5202	13.9%	5221	13.9%	2159	11.9%	2226	12.3%	7131	12.9%	7146	12.9%
Congestive heart failure	11,897	31.7%	12,028	32.1%	5114	28.2%	5177	28.6%	16,729	30.2%	16,615	30.0%
Diabetes	13,442	35.8%	13,565	36.1%	6731	37.1%	6753	37.2%	20,370	36.8%	20,298	36.7%
Hypertension	33,730	89.9%	33,816	90.1%	15,934	87.9%	15,991	88.2%	48,716	88.0%	48,780	88.1%
Renal disease	8479	22.6%	8508	22.7%	2892	16.0%	2984	16.5%	10,376	18.7%	10,392	18.8%
Myocardial infarction	4941	13.2%	4990	13.3%	1940	10.7%	2040	11.3%	6890	12.4%	6877	12.4%
Dyspepsia or stomach discomfort	8427	22.5%	8411	22.4%	3597	19.8%	3691	20.4%	11,843	21.4%	11,852	21.4%
Peripheral vascular disease	22,042	58.7%	22,245	59.3%	9669	53.3%	9867	54.4%	30,815	55.7%	30,831	55.7%
Peripheral artery disease	8717	23.2%	9076	24.2%	3633	20.0%	3707	20.4%	12,412	22.4%	12,567	22.7%
Transient ischemic attack	3384	9.0%	3395	9.0%	1338	7.4%	1344	7.4%	4342	7.8%	4373	7.9%
Coronary artery disease	19,294	51.4%	19,501	52.0%	8347	46.0%	8582	47.3%	26,481	47.8%	26,523	47.9%
*Baseline medication use*												
Angiotensin converting enzyme inhibitor	12,998	34.6%	13,084	34.9%	6859	37.8%	6841	37.7%	19,972	36.1%	20,044	36.2%
Amiodarone	3867	10.3%	3801	10.1%	1614	8.9%	1637	9.0%	4355	7.9%	4360	7.9%
Angiotensin receptor blocker	9532	25.4%	9538	25.4%	4478	24.7%	4603	25.4%	13,103	23.7%	13,042	23.6%
Beta blockers	21,347	56.9%	21,379	57.0%	9731	53.7%	9777	53.9%	29,724	53.7%	29,670	53.6%
H2-receptor antagonist	2728	7.3%	2797	7.5%	1208	6.7%	1232	6.8%	3800	6.9%	3822	6.9%
Proton pump inhibitor	12,520	33.4%	12,521	33.4%	5347	29.5%	5553	30.6%	17,089	30.9%	17,116	30.9%
Anti-platelets	8722	23.2%	8814	23.5%	3436	19.0%	3510	19.4%	11,334	20.5%	11,404	20.6%
Statins	22,711	60.5%	22,960	61.2%	10,449	57.6%	10,589	58.4%	31,640	57.2%	31,568	57.0%
*Inpatient admission*	14,935	39.8%	15,081	40.2%	6819	37.6%	6986	38.5%	23,133	41.8%	23,214	41.9%
*Patients on standard dose DOAC*	26,628	71.0%			14,496	80.0%			36,656	66.2%		
*Mean follow-up time (in days)*	230.3	211.3	281.3	260.0	257.0	265.9	285.6	264.7	275.8	265.7	284.0	262.7

CHA_2_DS_2_-VASc: congestive heart failure, hypertension, age ≥ 75 years, diabetes mellitus, prior stroke or transient ischemic attack or thromboembolism, vascular disease, age 65–74 years, sex category; HAS-BLED: hypertension, abnormal renal and liver function, stroke, bleeding, labile INRs (international normalized ratio), elderly, drugs, and alcohol; PSM: propensity score matching; SD: standard deviation

^a^As the INR value was not available in the data, a modified HAS-BLED score was calculated using a range of 0 to 8

### Stroke/SE and MB

**Fig. 2:**
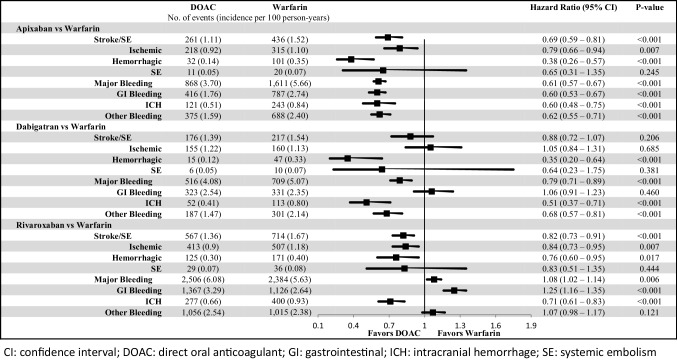
Incidence rate and hazard ratio of stroke/SE and major bleeding for propensity score-matched patients

Compared to warfarin, apixaban (HR: 0.69; 95% confidence interval [CI] 0.59–0.81, p < 0.001) and rivaroxaban (HR: 0.82; 95% CI 0.73–0.91, p < 0.001) were associated with a significantly lower risk of stroke/SE ; dabigatran (HR: 0.88; 95% CI 0.72–1.07, p = 0.206) was associated with a non-significantly lower risk of stroke/SE (Fig. 2). All DOACs were associated with a lower risk of hemorrhagic stroke versus warfarin.

Compared to warfarin, apixaban (HR: 0.61; 95% CI 0.57–0.67, p < 0.001), and dabigatran (HR: 0.79; 95% CI 0.71–0.89, p < 0.001) were associated with a significantly lower risk of MB, and rivaroxaban (HR: 1.08; 95% CI 1.02–1.14, p = 0.006) was associated with a higher risk of MB, mainly due to GI bleeding (Fig. 2). All DOACs were associated with a lower risk of ICH versus warfarin.

### NCO and MACE

**Fig. 3:**
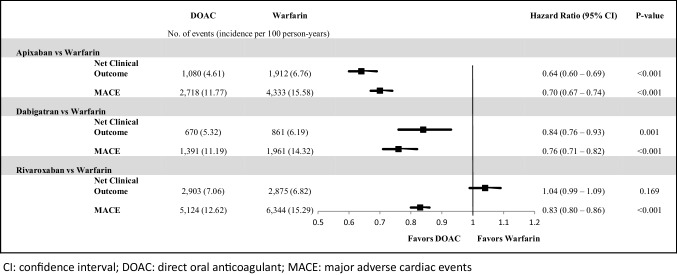
Incidence rates and hazard ratios of net clinical outcome and MACE for propensity score-matched patients

As a composite of stroke/SE and MB, the risk of NCO was significantly lower than warfarin for apixaban (HR: 0.64; 95% CI 0.60–0.69, p < 0.001) and dabigatran, (HR: 0.84; 95% CI 0.76–0.93, p = 0.001) but similar for rivaroxaban (HR: 1.04; 95% CI 0.99–1.09, p = 0.169) (Fig. 3).

Compared to warfarin, all DOACs were associated with a lower risk of MACE (apixaban: HR: 0.70; 95% CI 0.67–0.74, p < 0.001; dabigatran: HR: 0.76; 95% CI 0.71–0.82, p < 0.001; rivaroxaban: HR: 0.83; 95% CI 0.80–0.86, p < 0.001; Fig. 3).

### Sensitivity analysis

In the sensitivity analysis wherein the follow-up period was censored at 6 months, the results were consistent with the main analysis (Supplemental Table 1).

## Discussion

Using Medicare FFS data from 2012 to 2015, this study showed that compared to warfarin among elderly patients with NVAF, apixaban was associated with significant lower risks of stroke/SE, MB, NCO, and MACE. Dabigatran was associated with significantly lower risks of MB, NCO, and MACE as well as a numerically lower risk of stroke/SE. Rivaroxaban was associated with lower risks of stroke/SE and MACE, but higher MB and numerically higher NCO risks compared to warfarin.

The study results supplement RCT findings for apixaban, dabigatran, and rivaroxaban compared to warfarin and their corresponding age subgroup analyses [20–25]. In the RE-LY trial, patients (overall and ≥ 75 years) with 150 mg dabigatran had lower rates of stroke/SE and similar rates of MB compared to warfarin [20, 23]. In this real-world study among NVAF patients aged ≥ 65 years, 150 mg and 75 mg dabigatran showed numerically lower stroke/SE and significantly lower MB risks versus warfarin. Although NCO was not studied in the RE-LY trial’s elderly group, overall dabigatran and warfarin patient analysis demonstrated that compared to warfarin, 150 mg twice-daily dabigatran was associated with a non-significantly lower risk of net clinical benefit (a composite of stroke/SE, pulmonary embolism, MI, death, and MB) [20]. In this study, elderly dabigatran patients were associated with significantly lower NCO and MACE risks than warfarin patients.

In the ARISTOTLE trial, apixaban was associated with lower rates of stroke/SE, MB, and net clinical events (stroke/SE, MB, and all-cause death) compared to warfarin among all patients and patients aged ≥ 65 years [22, 25]. This study found consistent trends. In the ROCKET AF trial, rivaroxaban was associated with a non-inferior rate of stroke/SE and similar rate of MB compared to warfarin [21]. Among patients aged ≥ 75 years, 20 and 15 mg daily rivaroxaban showed a numerically lower risk of stroke/SE but a higher risk of MB compared to warfarin [24]. This study found similar trends between rivaroxaban and warfarin among patients aged ≥ 65 years. To the best of our knowledge, no previous studies have compared net clinical benefits between rivaroxaban and warfarin.

Several real-world studies have focused on effectiveness and safety comparisons between DOACs and warfarin in an elderly NVAF population [11, 26–29]. Our previous study of the elderly Medicare population from 2012 to 2014 consistent results of stroke/SE and major bleeding were found for the comparisons between DOACs and warfarin [11]. This study provides more recent and comprehensive analysis with updated data and added NCO and MACE outcomes. Using Medicare data from 2010 to 2012, Graham et al. [26] demonstrated that compared to warfarin, elderly NVAF dabigatran initiators (aged ≥ 65 years) were associated with lower risks of ischemic stroke, ICH, and death; similar risk of acute MI and MB; and a higher major GI bleeding risk. Our results (over an updated time-frame) showed consistent trends for ICH and GI bleeding. However, the ischemic stroke risk was similar, and the MB risk was lower for dabigatran versus warfarin patients in our study. Using Humana data, Deitelzweig et al. [27] found that NVAF patients aged ≥ 65 years with Medicare Advantage coverage who were treated with apixaban were associated with significantly lower risks of stroke/SE and MB compared to warfarin. This study noted consistent trends.

A few other real-world studies among DOACs and warfarin have provided comparative effectiveness and safety information by conducting subgroup analyses for age subgroups [30–32]. Using MarketScan and Optum data from 2010 to 2012, Seeger et al. showed that among patients aged 65–74 years, compared to warfarin, dabigatran was associated with similar risk for stroke and lower risk for MB; among those aged ≥ 75 years, dabigatran was associated with lower risk for stroke and similar risk for MB [30]. Using the MarketScan data from 2010 to 2014, Norby et al. [31] found that among patients aged ≥ 75 years, rivaroxaban was associated with a similar risk for ischemic stroke and MI, a lower ICH risk, and a higher GI bleeding risk compared to warfarin. Using a pooled dataset, Li et al. [32] demonstrated that among elderly patients, apixaban was associated with similar (65–74) to lower (≥ 75) stroke/SE risk and a lower (65–74 and ≥ 75) MB risk compared to warfarin. The comparisons between DOACs and warfarin in our study showed trends generally consistent with previous literature. However, more studies are needed to better understand effectiveness and safety profiles in elderly populations. Moreover, as DOAC use increases, further research will be necessary to assist in decision-making for such populations [33].

Despite growing evidence of improved safety with DOACs, warfarin is still widely used in high-risk NVAF populations [34]. Our study provides a current and comprehensive analysis comparing DOACs and warfarin regarding the risk of stroke/SE, MB, NCOs, and MACE among elderly US Medicare NVAF patients. Given the distinct clinical characteristics of the elderly NVAF population, the study results may add useful information to the literature to assist in disease management decision making.

This study has several limitations. Given its observational nature, confounding factors may have impacted the results. To control for potential confounders, a comprehensive list of baseline covariates was included in the PSM, including patient demographics and clinical characteristics. However, variables such as over-the-counter use of aspirin, serum creatinine/creatinine clearance, and laboratory test result values are not captured in the Medicare data. As claims data analysis, the study may also be subject to coding errors and inaccurate or incomplete clinical information. For example, treatments recorded based on prescription claims include no evidence of drug adherence. Moreover, since international normalized ratio values were not obtained, the quality of warfarin treatment could not be evaluated and the calculation for HAS-BLED score was modified. Moreover, proper dosage for DOACs based on age, renal function, and weight could not be assessed.

In summary, in the elderly Medicare population with NVAF, compared to warfarin, the DOACs were associated with a lower to similar risk of stroke/SE and MACE, but with varying comparative risks for MB and NCO.

## Electronic supplementary material

Below is the link to the electronic supplementary material.

Supplementary material 1 (DOCX 11 KB)
