# Supplementary material for: Comparative clinical outcomes between direct oral anticoagulants and warfarin among elderly patients with non-valvular atrial fibrillation in the CMS medicare population
Source: J Thromb Thrombolysis. 2019 Mar 28;48(2):240–9. doi: 10.1007/s11239-019-01838-5 (PMC6599494; doi:10.1007/s11239-019-01838-5)
Supplement: Supplementary file 1 — Supplementary material 1 (DOCX 11 KB) [file 11239_2019_1838_MOESM1_ESM.docx]

**Supplemental Table 1. Risk of Stroke/SE, Major Bleeding, and MACE Within 6 Months of Treatment Initiation**

|  | **Apixaban vs Warfarin** | **Dabigatran vs Warfarin** | **Rivaroxaban vs Warfarin** |
| --- | --- | --- | --- |
| **Stroke/SE** | 0.67 (0.56, 0.82) | 0.95 (0.74, 1.21) | 0.79 (0.68, 0.91) |
| **Major Bleeding** | 0.58 (0.52, 0.64) | 0.75 (0.65, 0.87) | 1.12 (1.04, 1.20) |
| **MACE** | 0.68 (0.64, 0.71) | 0.74 (0.68, 0.80) | 0.83 (0.79, 0.86) |
| **Net Clinical Outcome** | 0.60 (0.55, 0.66) | 0.82 (0.72, 0.92) | 1.05 (0.99, 1.12) |
